# Navigating Cardiotoxicity in Immune Checkpoint Inhibitors: From Diagnosis to Long-Term Management

**DOI:** 10.3390/jcdd12070270

**Published:** 2025-07-16

**Authors:** Simone Nardin, Beatrice Ruffilli, Pietro Costantini, Rocco Mollace, Ida Taglialatela, Matteo Pagnesi, Mauro Chiarito, Davide Soldato, Davide Cao, Benedetta Conte, Monica Verdoia, Alessandra Gennari, Matteo Nardin

**Affiliations:** 1 Division of Medical Oncology, Maggiore University Hospital, 28100 Novara, Italy; 2 Department of Internal Medicine and Medical Sciences (Dimi), School of Medicine, University of Genova, 16126 Genova, Italy; 3 Department of Translational Medicine, University of Piemonte Orientale, 28100 Novara, Italy; 4 Radiology Department, Maggiore University Hospital, 28100 Novara, Italy; 5 Department of Cardiology, Humanitas Gavazzeni Hospital, 24125 Bergamo, Italy; 6 Department of Experimental Medicine, Università degli Studi di Roma Tor Vergata, 00133 Roma, Italy; 7 Institute of Cardiology, ASST Spedali Civili, Department of Medical and Surgical Specialties, Radiological Sciences and Public Health, University of Brescia, 25121 Brescia, Italy; 8 Department of Biomedical Sciences, Humanitas University, Via Rita Levi Montalcini 4, Pieve Emanuele, 20090 Milan, Italy; 9 IRCCS Humanitas Research Hospital, Via Manzoni 56, Rozzano, 20089 Milan, Italy; 10 U.O. Clinica di Oncologia Medica, IRCCS Ospedale Policlinico San Martino, 16132 Genova, Italy; 11 Division of Cardiology, Nuovo Ospedale degli Infermi, ASL Biella, 13875 Biella, Italy; 12 Internal Medicine, Department of Medicine, ASST Spedali Civili di Brescia, Piazzale Spedali Civili 1, 25123 Brescia, Italy

**Keywords:** immune checkpoint inhibitors, cardiovascular toxicity, myocarditis, diagnosis, treatment, myocarditis

## Abstract

The advent of immune checkpoint inhibitors (ICIs) has revolutionized cancer treatment, significantly improving patient outcomes across multiple malignancies. Nonetheless, these therapies are associated with immune-related adverse effects, including cardiotoxicity, which remains a critical concern. This review provides a comprehensive analysis of ICI-related cardiotoxicity, encompassing its pathophysiological mechanisms, risk factors, diagnostic modalities, and management strategies. The onset of cardiotoxicity varies widely, ranging from acute myocarditis to long-term cardiovascular complications. Early identification through clinical assessment, biomarkers, and advanced imaging techniques is crucial for timely intervention. Management strategies include high-dose corticosteroids, other immunosuppressive agents, and supportive therapies, with a focus on balancing oncologic efficacy and cardiovascular safety. Additionally, rechallenging patients with ICIs following cardiotoxic events remains a complex clinical decision requiring multidisciplinary evaluation. As immunotherapy indications expand to include high-risk populations in a curative setting too, optimizing screening, prevention, and treatment strategies is essential to mitigate cardiovascular risks. A deep understanding of the molecular and clinical aspects of ICI-related cardiotoxicity will enhance patient safety and therapeutic decision-making, underscoring the need for ongoing research in this rapidly evolving field.

## 1. Introduction

Over the past two decades, the advent of immune checkpoint inhibitors (ICIs) has transformed the therapeutic landscape of oncology, offering unprecedented improvements in survival for a broad range of malignancies [1,2,3]. These agents function by “unlocking” immune inhibitory pathways, blocking key immune inhibitory pathways, such as cytotoxic T-lymphocyte-associated antigen 4 (CTLA-4), programmed cell death protein 1 (PD-1), and its ligand (PD-L1), thereby restoring and enhancing T-cell-mediated antitumor response [2,4]. While ICIs have shown significant efficacy, improving patients’ progression-free and overall survival, they are also associated with a broad spectrum of immune-related adverse events (irAEs) that can affect virtually any organ system.

Among these, cardiovascular (CV) toxicities are relatively uncommon, affecting about 1.14–5% of all irAEs, but they represent some of the most severe and potentially fatal complications [5]. ICI-related cardiotoxicity encompasses a heterogeneous group of conditions, including myocarditis, arrhythmias, pericarditis, and acute coronary syndromes [5,6,7]. Although the reported incidence of cardiac irAEs in clinical trials remains low, the associated mortality, particularly in myocarditis, is strikingly high [6]. Myocarditis occurs in approximately 0.06–1.1% of patients treated with ICIs, with mortality rates reaching 36–50%, particularly in those receiving combination regimens. Arrhythmias, including atrial fibrillation and conduction disturbances, are reported in approximately 2–3%, while pericardial disease affects roughly 0.7–7% of patients. Limited impact has been observed for acute coronary syndromes, occurring in up to 1% of cases [7,8,9,10,11,12,13]. Moreover, real-world data suggest that the true incidence may be underestimated, with some studies reporting rates as high as 12.5% at 12 months post-treatment initiation [7]. As the clinical use of ICIs continues to expand, a deeper understanding of the risk factors, pathophysiological mechanisms, and optimal management strategies for CV toxicities is urgently needed. This review aims to comprehensively analyze ICI-related cardiotoxicities, exploring pathophysiology, risk factors, diagnosis, and management strategies. The goal is to enhance understanding and improve clinical practices to maximize therapeutic benefits while minimizing CV risks.

## 2. Immune Checkpoint Inhibitors

**Figure 1:**
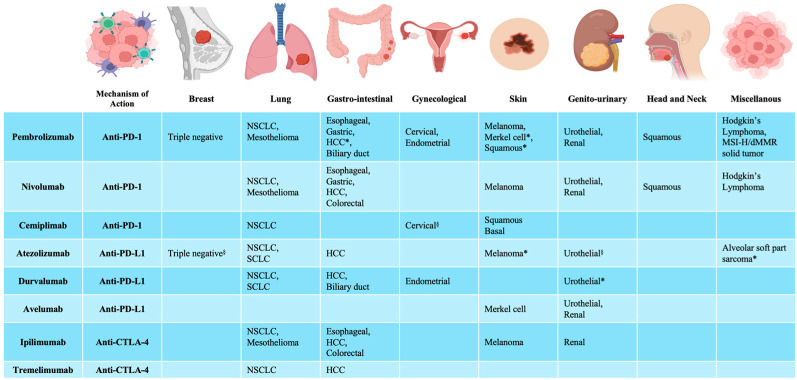
Immune checkpoint inhibitors. Food and Drug Administration (FDA) and European Medicines Agency (EMA) approvals for immune checkpoint inhibitors. * only approved by FDA; ^§^ only approved by EMA. NSCLC = non-small cell lung cancer; HCC = hepatocellular carcinoma; MSI-H = microsatellite instability-high; dMMR = mismatch repair deficient; SCLC = small cell lung cancer. Created with BioRender.com.

Immunotherapy has gained prominence as a transformative strategy in managing solid tumors, offering significant clinical advantages across various cancer types. Among the most impactful immunotherapeutic strategies, ICIs work by disrupting immune regulatory mechanisms, thereby enabling T-cells to more effectively identify and attack tumor cells [14]. Currently, ICIs are approved for a growing number of indications, with nearly half of all patients with metastatic cancer in high-income countries eligible for these therapies [15,16] (Figure 1). Beyond the metastatic setting, ICIs are increasingly being incorporated into neo-adjuvant and maintenance treatment regimens, and are often used in combination with other ICIs, cytotoxic chemotherapy, targeted agents, or biologics [17].

The immune checkpoints act as critical regulators of immune tolerance, preventing autoimmunity by downregulating T-cell activation [5,18]. However, during tumorigenesis, cancer cells exploit these pathways to evade immune surveillance, thereby promoting tumor progression [18,19,20]. By blocking these immune checkpoints, ICIs restore T-cell activity, enabling a more robust and sustained antitumor immune response [5].

**Figure 2:**
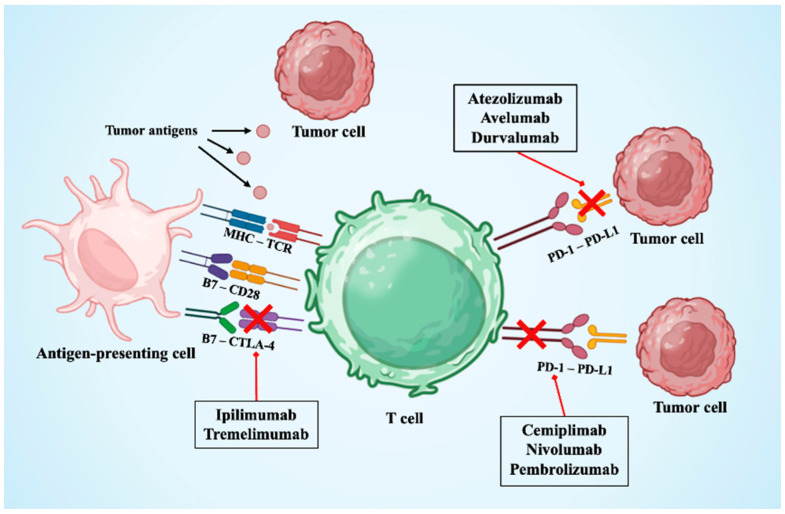
Immune checkpoint inhibitors’ targets. The figure shows the main targets of immune checkpoint inhibitors available. CTLA4 = cytotoxic T-lymphocyte antigen 4; MHC-TCR = major histocompatibility complex—T-cell receptor; PD-1 = programmed death-1; PD-L1 = programmed death-ligand-1. Created with BioRender.com.

Currently, multiple ICIs have received regulatory approval, targeting three main molecules: CTLA-4, PD-1, and PD-L1 (Figure 2). CTLA-4 is an inhibitory receptor expressed on T-cells that competes with CD28 for binding to the co-stimulatory molecules CD80 and CD86. This interaction inhibits T-cell receptor signaling and halts T-cell activation [18,21]. CTLA-4 inhibitors block CTLA-4 signaling on naïve T-cells and regulatory T-cells (Treg), resulting in enhanced T-cell priming and reduced peripheral tolerance. Gastrointestinal toxicities, such as colitis, are the most common: Treg depletion in the gut mucosa permits unchecked effector T-cell activation and mucosal injury [22,23]. Also, skin-related irAEs, including rash and vitiligo, have been described [24].

In contrast, the PD-1/PD-L1 pathway acts later in the immune response. PD-1 is expressed on activated T-cells, while its ligands, PD-L1 and PD-L2, are often upregulated on tumor cells and within the tumor microenvironment. Their interaction leads to T-cell exhaustion and immune evasion. PD-1 and PD-L1 inhibitors primarily act in peripheral tissues to restore exhausted CD8^+^ cytotoxic T-cells and Th1-polarized CD4^+^ helper T-cells [25]. Lung toxicities are among the most common serious irAEs, with PD-1 inhibitors showing pulmonary lymphoid infiltration [26]. Nephritis and cystitis have been described, while breast and head and neck cancer patients may experience autoimmune mastitis and mucosal inflammation [27]. Unlike conventional chemotherapy-related side effects, irAEs result from the disruption of immune homeostasis and the loss of peripheral tolerance, leading to off-target immune responses against healthy tissues. These events can affect virtually any organ system, with the skin, gastrointestinal tract, liver, and endocrine system being the most frequently involved.

The incidence of irAEs is reported in a significant proportion of patients undergoing ICIs, with the frequency and severity varying widely depending on the specific agent and treatment regimen, cancer type, and patient-specific factors. Overall, the incidence of irAEs is about 70% in patients treated with anti-PD1/PD-L1 monotherapy and approximately 80% in those receiving CTLA-4; combination regimens are associated with increased rates of adverse events, with common terminology criteria for adverse events (CTCAE)-defined grade 3 or higher toxicities occurring in up to 55% of patients, considering all type of toxicities, not only CV irAEs [20,28,29,30].

Most immune-related adverse events are mild to moderate. They can be successfully managed by delaying ICI administration or temporarily suppressing the immune system with corticosteroids or additional second-line immunosuppressants [4,31,32]. However, some cases progress to severe or life-threatening complications despite immunosuppressive treatments.

## 3. Cardiotoxicity Rationale and Onset

Among the less common but potentially fatal toxicities associated with ICIs, CV irAEs have emerged as an area of increasing concern. Cardiotoxicity related to ICIs includes a spectrum of conditions such as myocarditis, pericarditis, arrhythmias, and heart failure (HF) [9,12,32,33,34].

The estimated incidence of ICI-related cardiotoxicity in clinical trials remains low, ranging from 0.04% to 1.14%; however, real-world data suggest these figures may be underestimated owing to underreporting, nonspecific symptoms, and diagnostic difficulties [11,33,34,35].

Combination therapies with dual ICIs, such as one anti-CTLA-4 and one anti-PD-1/PD-L1antibodies, are associated with a notably higher risk of CV irAEs compared to monotherapy [10]. Meta-analyses and pharmacovigilance studies indicate a twofold to threefold increased risk of myocarditis, pericarditis, and fatal cardiac events in patients receiving combination therapy [11,28]. Palaskas et al. attributed this heightened cardiotoxicity to additive immune activation and intensified myocardial inflammation [36]. Moreover, the concurrent use of ICIs with other anticancer therapies, such as tyrosine kinase inhibitors, chemotherapy, or radiation therapy, may further potentiate CV toxicity by exacerbating systemic inflammatory responses and direct vascular damage [36,37].

### 3.1. Pathophysiology

The underlying pathophysiological mechanisms of ICI-related cardiotoxicity are complex and remain incompletely understood. Increasing evidence suggests these toxicities are predominantly immune-mediated, driven by disruptions in peripheral immune tolerance and subsequent activation of autoreactive T-cells. Molecular mimicry, wherein activated T-cells against tumor antigens also recognize structurally similar antigens expressed by cardiomyocytes, may lead to unintended immune responses against cardiac tissues [33].

Under normal physiological conditions, the heart is traditionally viewed as an immune-privileged organ, partly due to low basal expression of major histocompatibility complex molecules and the local presence of immunomodulatory ligands, including PD-L1 on cardiomyocytes. This immunological tolerance appears to be critically disrupted by ICIs, particularly PD-1/PD-L1 blockade: in their pivotal work, Moslehi et al. demonstrated that genetic deletion or pharmacologic inhibition of PD-1 leads to spontaneous myocarditis in murine models, characterized by diffuse CD8^+^ T-cell infiltration and myocardial necrosis [9,33,34,35].

A preclinical study by Axelrod et al. evaluated the role of α-myosin in a mouse model of ICI-related myocarditis [38]: it showed a substantial role in promoting highly inflamed cardiac T-cells at the time of death due to myocarditis, suggesting α-myosin-reactive T-cell receptors (TCRs) not only as a mechanistic insight but also as a potential diagnostic biomarker and therapeutic target for selective immune modulation.

Histological examinations of ICI-related myocarditis frequently reveal substantial infiltration by immune cells, particularly cytotoxic CD8^+^ T-cells and macrophages, with an evident absence of B-cell involvement, within the myocardium and the conduction system, often associated with myocyte injury and necrosis [33,34,35]. This supports the hypothesis that T-cell–mediated cytotoxicity is central to cardiac injury. Furthermore, clonal expansion of T-cells identified in both the tumors and myocardium of patients presenting with acute myocardial inflammation further supports this hypothesis [39].

### 3.2. Preclinical and Animal Studies

Preclinical studies have reinforced these findings, demonstrating that PD-1 knockout mice spontaneously develop autoimmune dilated cardiomyopathy, highlighting the critical regulatory role of PD-1 in preventing autoimmune myocardial damage [40]. Moreover, animal studies involving combined ICI treatments have shown significant T-cell infiltration into cardiac tissues, paralleling human pathology [41]. The upregulation of inflammatory cytokines can intensify local inflammation and promote cardiomyocyte apoptosis [9,12,33]. Additionally, the unique structural and electrophysiological properties of the heart render it particularly vulnerable to immune-mediated damage. Even minor inflammation or limited myocyte necrosis can enhance the risk of arrhythmias, advanced heart block, or sudden cardiac death [33].

### 3.3. Non-Immune ICI Toxicities

While the prevailing paradigm attributes ICI-associated cardiotoxicity to dysregulated immune activation, non-immune off-target toxicities may also contribute. For example, PD-1 and PD-L1 are expressed not only on immune cells but also on endothelial cells and cardiomyocytes, where they exert anti-apoptotic and homeostatic effects via MAPK and PI3K/Akt signaling, provoking direct endothelial dysfunction or cardiomyocyte stress [42]. Moreover, a recent proteomic analysis of human ICI-related myocarditis identified perturbations in mitochondrial metabolic proteins, suggesting that altered mitochondrial bioenergetics may also play a direct, non-immune role in myocardial injury [43]. Although histopathologic studies in clinically confirmed myocarditis almost always reveal immune infiltration, other CV complications can share non-immune mechanisms.

## 4. Risk Factors Related to Cardiotoxicity

ICIs significantly enhance cancer treatment outcomes but carry an increased risk of severe CV irAEs, including myocarditis, pericarditis, arrhythmias, and vasculitis. Comprehensive knowledge of patient-specific and treatment-related risk factors is essential to identify high-risk populations and implement preventive strategies effectively.

### 4.1. Demographics

Sex, race, and tobacco use influence susceptibility to ICI-related cardiotoxicity. Females generally exhibit stronger immune responses, leading to slightly higher irAEs, yet males experience more severe myocarditis [8,44], likely due to sex-based immune and hormonal differences [8,43]. Emerging data suggest African American patients might have a higher incidence of myocarditis, possibly reflecting immune variations, differential checkpoint molecule expression, and CV health disparities [11,45,46,47]. Tobacco use exacerbates CV risk by promoting inflammation and oxidative stress, potentially heightening cardiotoxicity risk during ICI therapy; thus, smoking cessation is crucial for risk mitigation [37,48,49,50].

### 4.2. Pre-Existing Cardiovascular Conditions

Pre-existing CV disease, including ischemic heart disease, HF, arrhythmias, and hypertension, has been consistently associated with an elevated risk of ICI-related cardiotoxicity. Mahmood et al. reported that approximately 46% of patients developing myocarditis during ICI therapy had a prior CV history, particularly hypertension and coronary artery disease [8]. Similarly, Lyon et al. emphasized that patients with underlying cardiac disease have a heightened vulnerability to myocarditis, pericarditis, and accelerated atherosclerosis following ICI exposure [48]. Additional retrospective analyses have identified hypertension, diabetes mellitus, and prior myocardial infarction as independent predictors of major adverse CV events during ICI therapy [36,37]. Similarly, common comorbidities as dyslipidemia, chronic kidney disease, and obstructive sleep apnea have been linked to a greater risk of developing severe immune-related CV events [37], beyond the enhanced overall CV risk [51,52,53]. Obesity promotes a chronic low-grade inflammatory state, endothelial dysfunction, and dysregulated immune responses [54,55], which may amplify the risk of myocarditis, pericarditis, and vascular inflammation under ICIs. A recent meta-analysis has demonstrated a relationship between higher BMI, both as overweight and obesity, and increased risk of irAEs in patients on ICI therapies [56].

### 4.3. Underlying Autoimmune Diseases

Patients with pre-existing autoimmune diseases, including rheumatoid arthritis, systemic lupus erythematosus, myasthenia gravis, and sarcoidosis, are at a markedly increased risk of developing irAEs during ICI therapy [47,57,58]. Autoimmune conditions predispose to heightened immune activation and loss of tolerance, mechanisms that are believed to amplify the risk of myocarditis and other cardiac complications. Salem et al. observed that myocarditis incidence is particularly elevated among patients with concomitant skeletal myositis or myasthenia gravis, with reported mortality rates exceeding 50% in dual-affected individuals [11]. Furthermore, patients with baseline autoimmune diseases often present with overlapping systemic inflammation that complicates the diagnosis and management of ICI-related cardiotoxicity [58]. Early CV evaluation and multidisciplinary care are strongly recommended for this vulnerable subgroup.

### 4.4. Tumor Type

The type of malignancy treated with ICIs appears to influence the incidence and phenotype of CV irAEs. Melanoma has been associated with an increased incidence of myocarditis, possibly due to intense T-cell activation against shared antigens between melanoma cells and myocardial tissue [47,59]. Lung cancer patients exhibit a relatively high incidence of pericardial disease, including pericarditis and pericardial effusions, likely linked to tumor proximity and local inflammatory responses within the thoracic cavity [37,59]. Additionally, patients with renal cell carcinoma and head and neck squamous cell carcinoma have been reported to develop rare vasculitis during ICI therapy, suggesting tumor-specific modulation of immune responses [11]. These observations highlight the necessity of personalized cardiac risk assessment based on tumor type and its associated immunological profile.

### 4.5. Genetic Factors

Genetic predisposition is increasingly recognized as a determinant of susceptibility to ICI-related cardiotoxicity. Single-nucleotide polymorphisms (SNPs) in immune checkpoint genes, including CTLA-4, PD-1, and PD-L1, may influence individual immune activation thresholds and the risk of autoimmune manifestations [60]. Recent mechanistic studies have implicated α-myosin-reactive T-cells as mediators of myocarditis following immune checkpoint blockade, with shared antigenic epitopes between tumor and cardiac tissues driving cross-reactive T-cell responses [38]. Whole-exome sequencing has identified germline variants associated with susceptibility to autoimmune myocarditis that overlap with variants found in patients developing myocarditis [38,61]. Understanding genetic predisposition may eventually enable personalized screening strategies for cardiotoxicity risk in patients considered for ICI therapy.

## 5. Cardiovascular Complications

Immune-related CV toxicities have emerged in the last years, evolving from focusing initially on rare but fatal ICI-related myocarditis with cardiogenic shock to more common, with low mortality rate complications, including non-fatal myocarditis, pericarditis, arrhythmias, HF, and coronary artery disease. The events usually take place early during treatment; however, the timing of onset may vary depending on the specific cardiac manifestation and the therapeutic regimen employed. More than 70% of myocarditis cases develop within the first six weeks, and ICI-related myocarditis frequently appears during the first few weeks of therapy, with a median onset reported between 17 and 34 days [34,35]. Although early onset is most common, delayed presentations have also been reported, including cases that arise several months after therapy initiation [34,35]. This variability in onset timing emphasizes the importance of prolonged clinical surveillance and monitoring throughout and even beyond the active treatment phase to ensure timely detection and management of potentially life-threatening cardiac complications. Baseline assessment includes standard recommendations plus the dosage of troponin and brain natriuretic peptide (BNP), together with echocardiogram among high-risk patients: they are subjects receiving dual ICI, combination ICI and cardiotoxic therapy, prior ICI-related non-CV events, and prior cancer therapy-related cardiac dysfunction or CV disease. At each of the first four cycles of oncological therapy, an electrocardiogram (ECG) and troponin assessment are recommended. Moreover, regular lipid and glycemic profile assessments are suggested. Long-term surveillance includes BNP dosage and echocardiogram, if required, every 6–12 months. Individualized modification can be made according to patients’ oncological and cardiological journey [62,63].

### 5.1. Myocarditis

ICI-related myocarditis is a rare but potentially fatal complication of cancer immunotherapy, with reported incidences between 0.09% and 1.14%, which can nearly double with combination therapies using CTLA-4 and PD-1 inhibitors [8,11,36,57]. Increased reporting in global databases such as WHO’s VigiBase and the Food and Drug Administration (FDA) Adverse Event Reporting System indicates growing recognition and possibly broader ICI use. Myocarditis typically occurs within the first month of therapy, though delayed cases exist, and is often associated with other irAEs such as myositis and hepatitis, indicating a more severe prognosis [64,65].

Severe, fulminant myocarditis generally presents early, especially following combination therapy, with mortality rates as high as 67%, compared to approximately 36% with monotherapy [33,34,35]. Histopathological studies of affected myocardium commonly reveal dense infiltration by T cells and macrophages [10,66].

**Figure 3:**
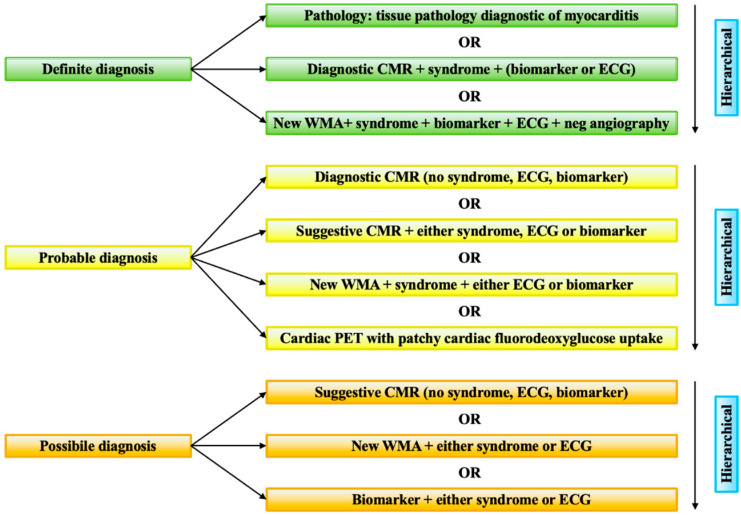
Bonaca’s criteria. The figure reports the criteria for the diagnosis of myocarditis in patients on therapeutics for cancer suggested by Bonaca et al. [67]. CMR = cardiac magnetic resonance; ECG = electrocardiogram; PET = positron emission tomography; WMA = wall motion abnormality.

**Figure 4:**
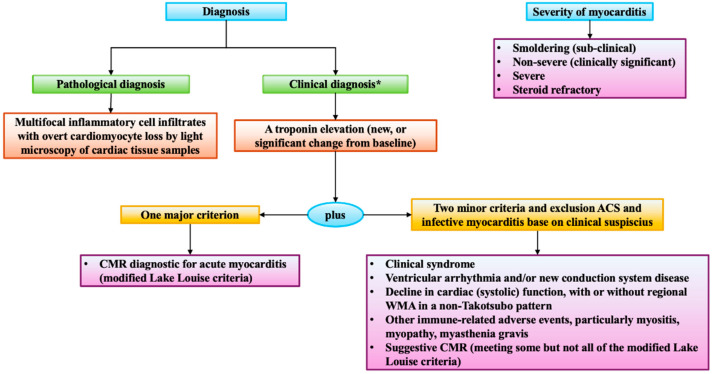
IC-OS criteria. The diagram shows the criteria for the diagnosis of myocarditis in patients on therapeutics for cancer proposed by the International Cardio-Oncology Society (IC-OS) [69]. * Clinical diagnosis should be confirmed with CMR or endomyocardial biopsy if possible and without causing delays in treatment. CMR = cardiac magnetic resonance; WMA = wall motion abnormalities.

Diagnosis of ICI-related myocarditis relies on integrating clinical presentation with biochemical, ECG, imaging, and pathological findings. Myocarditis should be diagnosed in acute cardiac conditions without an alternative primary diagnosis, such as acute coronary syndrome. Clinical manifestations show a wide range from asymptomatic cardiac biomarker elevations to cardiogenic shock. Bonaca et al. [67] have proposed a general framework for consideration of myocarditis with a three-tier category of definite, probable, and possible diagnosis of ICI-related myocarditis (Figure 3). Endomyocardial biopsy is the gold standard, especially in ambiguous or severe cases, showing lymphocytic infiltration and myocardial necrosis [68], although obtaining the sample is often challenging. The International Cardio-Oncology Society (IC-OS) has refined the diagnostic criteria [69], underscoring the importance of cardiac enzymes to suspect the acute damage in order to improve the sensitivity and reduce the risk of losing time in the diagnosis and treatment (Figure 4). In fact, serum biomarkers such as cardiac troponins and brain natriuretic peptide (BNP) are sensitive indicators of myocardial injury and dysfunction, with elevated troponins having high diagnostic value, despite limited specificity [58]. ECG abnormalities, including conduction disorders and arrhythmias, are commonly observed and signify a poor prognosis [70].

**Figure 5:**
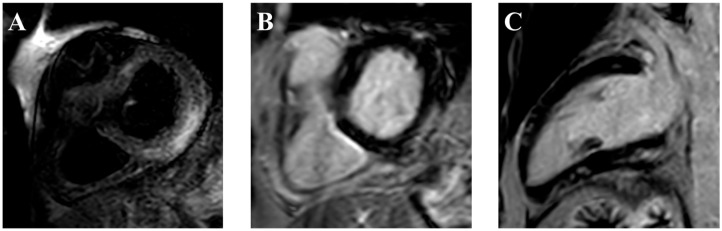
Cardiac magnetic resonance of an ICI-related acute myocarditis. The figure shows myocardial involvement. In particular, the short tau inversion recovery (STIR) image (**A**) reveals mesocardial hyperintensity in the basal inferior and inferolateral walls. Correspondingly, late gadolinium enhancement (LGE) images in the short-axis (**B**) and 2-chamber (**C**) views demonstrate subepicardial involvement of the same regions. Additionally, patchy subepicardial enhancement is observed in the mid-ventricular and apical anterior wall, as well as the mid-ventricular inferior wall. The radiological findings are consistent with myocarditis. ICI = immune checkpoint inhibitor.

Cardiac magnetic resonance (CMR) is the non-invasive imaging gold standard, strongly recommended when myocarditis is suspected due to its superior resolution and tissue characterization capabilities. The revised Lake Louise criteria emphasize advanced T1- and T2-mapping as critical diagnostic tools, the T1 for fibrosis and edema, while T2 for acute edema and inflammation [71]. The revised Lake Louise criteria have remarked on the importance of mapping alterations in the diagnosis of myocarditis by considering them a major criterion [31,72,73]. Late gadolinium enhancement (LGE) is less frequent than in viral myocarditis but present in up to 80% of ICI-related cases, typically involving the mid-wall septum [73,74] (Figure 5). A retrospective analysis of 79 patients with ICI-related myocarditis and available T1- and T2-mapping data showed that 100% of patients met at least one of the revised Lake Louise criteria and 48% met both T1 and T2-based criteria, with a pronounced diagnostic role for T1 mapping [75].

**Figure 6:**
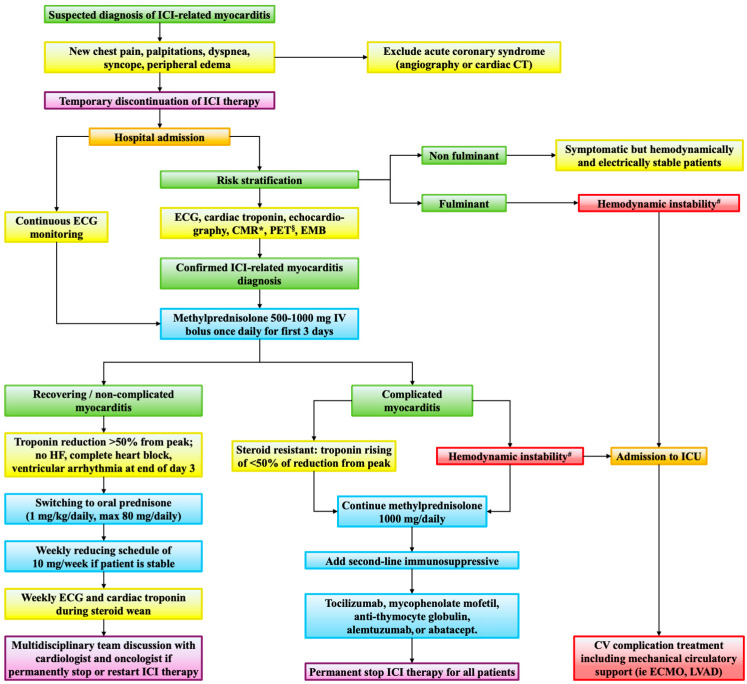
Diagnostic-therapeutic algorithm for immune checkpoint inhibitor-related myocarditis. The algorithm integrates the indications for the diagnosis and treatment of immune checkpoint inhibitors-related myocarditis suggested by the European Society of Cardiology (ESC) and the European Society of Medical Oncology (ESMO). * including T1 and T2 mapping, T2-weighted short tau inversion recovery (STIR), and late gadolinium enhancement. ^§^ using either [18F]-fluorodeoxyglucose (18FDG) or [68Ga]-DOTAT-octreotide. ^#^ hemodynamic instability includes acute decompensated heart failure, cardiogenic shock, non-invasive or invasive ventilation, complete heart block, and/or ventricular arrhythmia. CMR = cardiac magnetic resonance; CT = computed tomography; CV = cardiovascular; ECG = electrocardiogram; ECMO = extracorporeal membrane oxygenation; EMB = endomyocardial biopsy; HF = heart failure; ICI = immune checkpoint inhibitor; ICU = intensive care unit; IV = intravenous; LVAD = left ventricular assist device; PET = positron emission tomography.

Management strategies involve immediate cessation of ICI therapy and initiation of high-dose corticosteroids as the cornerstone of immunosuppressive therapy, typically 1000 mg of methylprednisolone intravenously daily for the first three days [76,77,78]. Patients judged responsive based on troponin reduction can start steroid tapering. For refractory cases, additional immunosuppressive agents, including mycophenolate mofetil, intravenous immunoglobulins, tacrolimus, infliximab, abatacept [59], alemtuzumab [79], and anti-thymocyte globulin [80] have been employed, although data supporting their use are primarily derived from case series and clinical experience [81,82]. Hemodynamic support, management of arrhythmia, and HF treatment following current guidelines are integral to patient care. Multidisciplinary management involving cardio-oncologists, cardiologists, internist immunologists, and oncologists is recommended to optimize patient outcomes [63]. Figure 6 shows the integrated algorithm for diagnosis and treatment proposed by the Heart Failure Association (HFA) of the European Society of Cardiology (ESC) and the European Society of Medical Oncology (ESMO) [81,82]. A recent risk score for major cardiotoxic events has been proposed, aiming to better stratify patients with ICI-related myocarditis. Results are of interest even if with modest overall performance, but considering its low-prevalence and high-mortality features, it can represent a useful tool during clinical management [83].

### 5.2. Pericarditis

Pericarditis has been reported as a potential CV complication associated with the use of ICIs in oncological therapies [84,85]. In a large retrospective cohort study, Gong et al. demonstrated that patients treated with ICIs exhibited a more than fourfold higher risk of developing pericardial disease compared with non-ICI-treated controls, reporting a hazard ratio (HR) of 4.37, with a 95% confidence interval (CI) of 2.09–9.14 [85]. Complementing these findings, a systematic review by Mudra et al. reported that pericardial disease typically developed after a median of four cycles of ICI therapy, with lung cancer representing the predominant underlying malignancy (81% of cases) [84]. Clinical presentation was often nonspecific, with dyspnea and chest pain being the most common symptoms, while classical diagnostic features such as ECG changes or pericardial rubs were infrequent. Notably, cardiac tamponade occurred in 41% of cases, necessitating pericardiocentesis in 68% of affected patients [84]. The presence of malignant cells in pericardial fluid, reported in a substantial proportion of cases, further complicates the distinction between malignant and immune-mediated pericardial effusions. Current management strategies predominantly involve ICI discontinuation, corticosteroids, and supportive care, although standardized diagnostic and therapeutic approaches remain lacking [11,84].

### 5.3. Arrhythmias

Arrhythmias are an emerging complication in patients undergoing ICIs therapy, often occurring early during treatment. Surveillance is crucial, as new-onset atrioventricular (AV) block, bundle branch block, or tachyarrhythmias may signal underlying other CV complications [81]. Atrial fibrillation is the most frequently reported arrhythmia (30% of cases), followed by ventricular tachyarrhythmias (27%) and conduction disorders (17%), the latter associated with a significantly higher CV mortality (80% vs. 16%) [58]. Notably, arrhythmias may develop even in the absence of myocarditis, potentially due to immune-mediated injury to conduction tissues, systemic inflammation, or myocardial metastases. Diagnostic work-up should include cardiac biomarkers, echocardiography, ECG telemetry, and in selected cases, CMR or endomyocardial biopsy [86]. Management focuses on treating the underlying cause, controlling the arrhythmia, and adjusting anti-cancer therapies through a multidisciplinary approach. Given the potential for late arrhythmic events, prolonged CV follow-up is recommended for cancer survivors treated with ICIs.

### 5.4. Heart Failure

HF is increasingly recognized in ICI-treated patients, typically after at least 6 months after therapy initiation. It has been reported across all ICI classes, with a potential predilection for patients with lung cancer treated with PD-1 inhibitors or those with pre-existing cardiac dysfunction. Some authors have proposed the term non-inflammatory left ventricular dysfunction (NILVD) to indicate this condition, underscoring the difference with myocarditis [81,87]: NILVD patients often have normal troponin levels, lack myocardial inflammation on CMR imaging, and exhibit few concurrent irAEs. In a retrospective analysis of 2647 patients started on ICI therapy, 15 patients of 89 experiencing CV irAEs showed left ventricular dysfunction without myocarditis, with a median time of onset of 26 weeks [87]. Diagnosis requires careful exclusion of other causes of left ventricular dysfunction, including acute coronary syndromes and myocarditis, using biomarkers, echocardiography, and, when appropriate, CMR [63]. Management follows standard HF therapy, including beta-blockers, renin–angiotensin–aldosterone system inhibitors, and diuretics, while immunosuppressive therapy such as steroids is not indicated [88]. In addition, the decision to continue versus interrupt ICI therapy depends upon the severity of the HF syndrome. The International Cardio-Oncology Society (IC-OS) proposed standardized definitions of cancer therapy-related cardiac complications [69]. Specifically, ICI therapy should be interrupted if patients present severe symptomatic or asymptomatic HF featured by a left ventricular ejection fraction < 40%, or symptomatic HF complicated by arrhythmia or cardiogenic shock. Mild cases are allowed to continue on ICIs. Multidisciplinary team discussion involving cardiology and oncology is recommended [63].

### 5.5. Vasculitis

Vasculitis is an uncommon but serious irAEs, affecting vessels of various sizes and leading to potential end-organ damage. In a large pharmacovigilance study, Salem et al. identified 82 vasculitis cases associated with ICIs, particularly temporal arteritis and polymyalgia rheumatica, with a higher incidence in males and a median onset of 55 days after therapy initiation [11]. Temporal arteritis, which can result in vision loss, was significantly over-reported in patients treated with ipilimumab for melanoma. In a multicenter European study, 67% of 27 patients with large vessel vasculitis met criteria for giant cell arteritis; 14.8% experienced visual impairment [89]. A case report also described eosinophilic temporal arteritis following atezolizumab in a patient with pre-existing eosinophilia, emphasizing the broad spectrum of vasculitis manifestations with ICIs [90]. Prompt recognition and initiation of immunosuppression are critical to avoiding irreversible complications such as blindness.

### 5.6. Atherosclerosis

Emerging data increasingly support an association between ICI therapy and atherosclerotic CV disease, given the growing importance of the immune system in its development [91,92]. Preclinical studies in Apoe−/− and Ldlr−/− mouse models show that CTLA-4 or PD-1 deficiency promotes plaque progression via enhanced T-cell–mediated inflammation and macrophage infiltration [93,94]. Clinically, ICI-treated patients exhibit accelerated atherosclerotic plaque development and destabilization, as evidenced by increased non-calcified plaque burden on CT imaging and elevated fluorodeoxyglucose (FDG) uptake on positron emission tomography (PET) scans [95,96]. In a pivotal retrospective cohort study, ICI therapy was associated with a threefold higher risk of myocardial infarction, ischemic stroke, or coronary revascularization compared to matched controls (HR 3.3; 95% CI: 2.0–5.5). Additionally, a case-crossover analysis demonstrated a 4.8-fold increased incidence of atherosclerotic events post-ICI initiation [97]. These findings are further supported by a meta-analysis of randomized trials showing elevated risk for myocardial infarction and cerebral ischemia in ICI-treated patients [98]. Despite robust experimental evidence and increasing clinical data, the role of immune checkpoint pathways in human atherosclerosis remains incompletely understood, warranting further longitudinal and mechanistic studies [99].

## 6. Immunotherapies Rechallenge

Reintroducing ICIs after the occurrence of irAEs is a growing area of clinical interest and a debated issue in oncology, particularly in patients with sustained clinical benefits and limited treatment options. The decision to resume therapy is multifactorial and depends on the type, grade, and reversibility of the initial irAEs, along with the patient’s oncologic status and the availability of other treatment options.

Current clinical guidelines suggest that ICI rechallenge may be appropriate for selected patients who have recovered from low-grade irAEs with a grade ≤2, according to CTCAE v5.0, especially those affecting skin, endocrine glands, or the gastrointestinal tract [31,82,100]. In contrast, rechallenge is generally discouraged in patients who have experienced severe or life-threatening irAEs, particularly those involving the CV or central nervous systems, due to the higher risk of recurrence and associated mortality [31,63,82,100].

Recent studies report that recurrence rates following ICI rechallenge range from approximately 28% to 43%, with nearly half of recurrent events being similar in intensity or milder compared to the initial presentation [101,102]. These rates appear to vary depending on the specific type of irAEs, underscoring the heterogeneity in underlying mechanisms and clinical severity across organ systems. Specifically, in the case of ICI-related myocarditis, recurrence upon rechallenge has been reported in approximately 37.5% of cases [102].

Several case reports have described real-world experiences of ICIs rechallenge following irAEs, providing insight into feasibility, safety, and outcomes. Otherwise, there have been only a few reports that have explored rechallenge after CV irAEs [103,104,105,106,107,108]. A case report documented a patient with prior fulminant myocarditis who successfully underwent rechallenge with different ICI regimens, highlighting the potential for safe rechallenge under strict monitoring protocols [103]. Similarly, another case described a patient with pembrolizumab-related myocarditis presenting as torsades de pointes, who was safely rechallenged with pembrolizumab after a mild myocarditis episode, suggesting that rechallenge may be feasible in cases of mild myocarditis without left ventricular dysfunction [104]. These reports indicate that ICI rechallenge can be considered in highly selected patients with prior cardiac events, provided there is a significant oncological benefit and strict CV monitoring. However, real-world evidence of rechallenge after ICI-related myocarditis revealed a high recurrence rate, underscoring the risk and the need for extreme caution when considering rechallenge in this setting [102]. Otherwise, a case report described a case of myocarditis recurrence following ICI-rechallenge; nevertheless, therapy was pursued with a favorable outcome, suggesting that rechallenge can be considered in highly selected patients with careful observation and timely intervention, especially in those without other treatment options [105].

As the use of ICIs continues to expand and more patients experience prolonged survival or durable responses, the question of whether, when, and how to safely resume therapy after CV irAEs has become increasingly relevant. In the absence of specific recommendations, rechallenge after CV irAEs should be approached through a comprehensive and individualized risk-benefit evaluation [100,106,108,109]. First, it must be considered the usefulness of rechallenge and the existence of alternative options. Moreover, the ICI regimen used for rechallenging should be carefully selected, often favoring monotherapy with PD-1/PD-L1 inhibitors over combination regimens, which are associated with a higher risk of recurrence and mortality. Second, patients’ characteristics and biological factors, such as comorbidities and performance status, are crucial elements. Third, the severity and clinical course of the initial cardiac toxicity play a pivotal role: rechallenge is generally contraindicated in cases of high-grade myocarditis (grade ≥ 2), especially those associated with arrhythmias, HF, or hemodynamic instability. In contrast, patients with mild, asymptomatic, or fully resolved cardiac events, and without structural or functional sequelae, may be potential candidates for cautious retreatment. Additionally, time to resolution and response to immunosuppressive therapy are also key considerations; indeed, complete clinical recovery and normalization of cardiac biomarkers, imaging, and ECG findings should be confirmed before reinitiating ICIs. This decision-making process should be guided by a multidisciplinary team, including cardio-oncology specialists [107,110]. A personalized risk-benefit assessment should consider the patient’s oncologic status, availability of alternative therapies, life expectancy, and the potential benefit of continued immunotherapy [101,107,109,110]. In selected cases, rechallenge may be accompanied by prophylactic measures and intensified monitoring protocols, including serial troponin levels, ECGs, and echocardiograms during the first weeks of retreatment. Finally, shared decision-making with the patient is critical, as the risk of recurrent or more severe cardiac events cannot be excluded.

## 7. Future Perspectives

As stated before, the use of ICI expands across various cancer types with progress through clinical trials, and an increasing number of patients benefit from these innovative immunotherapies. However, the widespread adoption of ICIs is accompanied by the risk of immune-related adverse events, and ICI-related cardiotoxicity, though relatively rare, is often severe and potentially life-threatening when it manifests clinically [48]. Salem et al. reported a pharmacovigilance analysis from WHO and FDA safety databases, revealing not only a disproportionately high reporting of myocarditis among ICI recipients but also a striking early mortality rate approaching 50%, often within the first 30 days [111], underscoring both the limitations of spontaneous adverse event reporting systems and the urgent need for amelioration of strategies of surveillance and treatment.

- Surveillance during ICI induction. Considering that 40–80% of those events occurred within the first month of ICI therapy, and 50% of myocarditis resulted in fatal outcomes, improvement in the first month of therapy would be crucial. Beyond the routine follow-up in high-risk patients before each ICI cycle, immediate cardio-oncology evaluation should not be delayed upon new ECG or biomarker changes [67,112,113]. Gong et al. proposed a surveillance strategy for patients undergoing ICIs [114]. Additionally, new biomarkers such as single-nucleotide polymorphism in miR-146a and miR-34 seem to predict irAEs in patients treated with ICIs, including cardiac toxicity [115,116,117,118,119].
- TCR profiling as predictive markers. Monitoring peripheral α-myosin-reactive T-cells may identify patients at increased myocarditis risk. Won et al., in a murine model, showed that PD-1 and CTLA-4 blockade led to the expansion and cardiac infiltration of α-myosin–specific CD8^+^ T cells, directly implicating them in the pathogenesis of myocardial injury [120]. These findings support the notion that loss of peripheral tolerance to cardiac-restricted antigens, such as α-myosin, is a central mechanism driving ICI-related cardiotoxicity and may offer a rationale for developing antigen-targeted therapeutic strategies.
- Neutrophil-to-lymphocyte ratio as an early myocarditis risk flag. A recent publication by Xue et al. evaluated the predictive value of the neutrophil-to-lymphocyte ratio in ICI-related myocarditis in a cohort of 146 patients affected by non-small cell lung cancer [121]. Among the 11.64% of patients who developed ICI-related myocarditis, an elevation in neutrophil-to-lymphocyte ratio ≥3.25 was reported as the most significant indicator of event occurrence (HR 11.094; 95% CI, 3.186–38.631; *p* < 0.001).
- Longitudinal cardio-oncology monitoring. The long-term consequences of ICI-related myocarditis remain unknown. The potential role of myocarditis as a “second hit” in genetically or otherwise predisposed individuals, potentially leading to cardiomyopathy (including, but not limited to, dilated cardiomyopathy), is receiving increasing attention [122,123]. However, no data are currently available regarding this mechanism in the context of ICI-related myocarditis. Likewise, there is no evidence on whether ICI-related myocarditis can act as a trigger for the development of cardiomyopathy, nor is there consensus on the optimal duration of follow-up for affected patients.

## 8. Conclusions

In conclusion, ICIs have significantly improved cancer treatment outcomes but are associated with rare yet severe cardiotoxicities, including myocarditis, pericarditis, arrhythmias, HF, vasculitis, and accelerated atherosclerosis. Early recognition through enhanced screening protocols and multidisciplinary management is crucial for timely diagnosis and intervention, balancing oncological efficacy and CV safety. The decision to rechallenge patients with ICIs after cardiotoxic events demands careful individual risk-benefit evaluation, emphasizing personalized strategies and vigilant monitoring. Further research into predictive biomarkers, underlying mechanisms, and targeted therapeutic approaches is essential to mitigate CV risks, optimize patient outcomes, and support safe integration of ICIs into broader oncology practice.
